# Supplementary material for: High-efficient fabrication of core-shell-shell structured SiO2@GdPO4:Tb@SiO2 nanoparticles with improved luminescence
Source: R Soc Open Sci. 2020 May 27;7(5):192235. doi: 10.1098/rsos.192235 (PMC7277279; doi:10.1098/rsos.192235)
Supplement: Supporting information [file rsos192235supp1.doc]

**[Supporting information]**

**High-efficient fabrication of core-shell-shell structured** **SiO_2_@GdPO_4_:Tb@SiO_2_ nanoparticles with improved luminescence**

He Bai^a^, Yunjiang Yang^a^, Jinrong Bao^a^,*, Anping Wu^a^, Yan Qiao^a^, Xueyuan Guo^a^, Mingyuan Wang^a^, Wenxian Li^a^, Ying Liu,^a^ & Xiaowei Zhu^b^

a. Inner Mongolia Key Laboratory of Chemistry and Physics of Rare Earth Materials, College of Chemistry and Chemical Engineering, Inner Mongolia University, Hohhot, 010021, China.

b. College of Pharmacology, Inner Mongolia Medical University, Hohhot 010110, China.

**
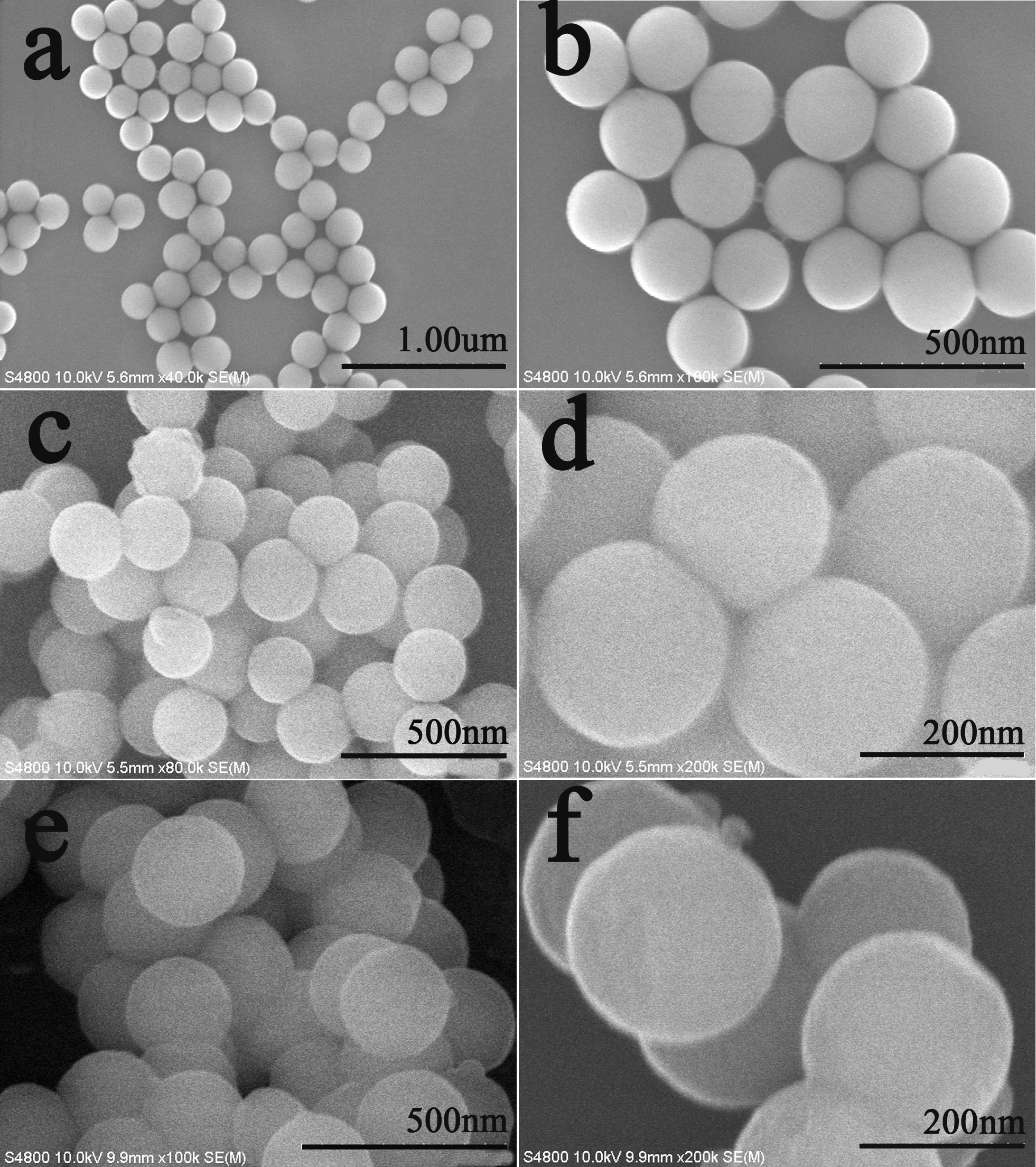
**

**Fig. S1.** FE-SEM images of the products: (a,b) SiO_2_, (c,d) SiO_2_@GdPO_4_:Tb, (e,f) SiO_2_@GdPO_4_:Tb@SiO_2_ nanoparticles.

**
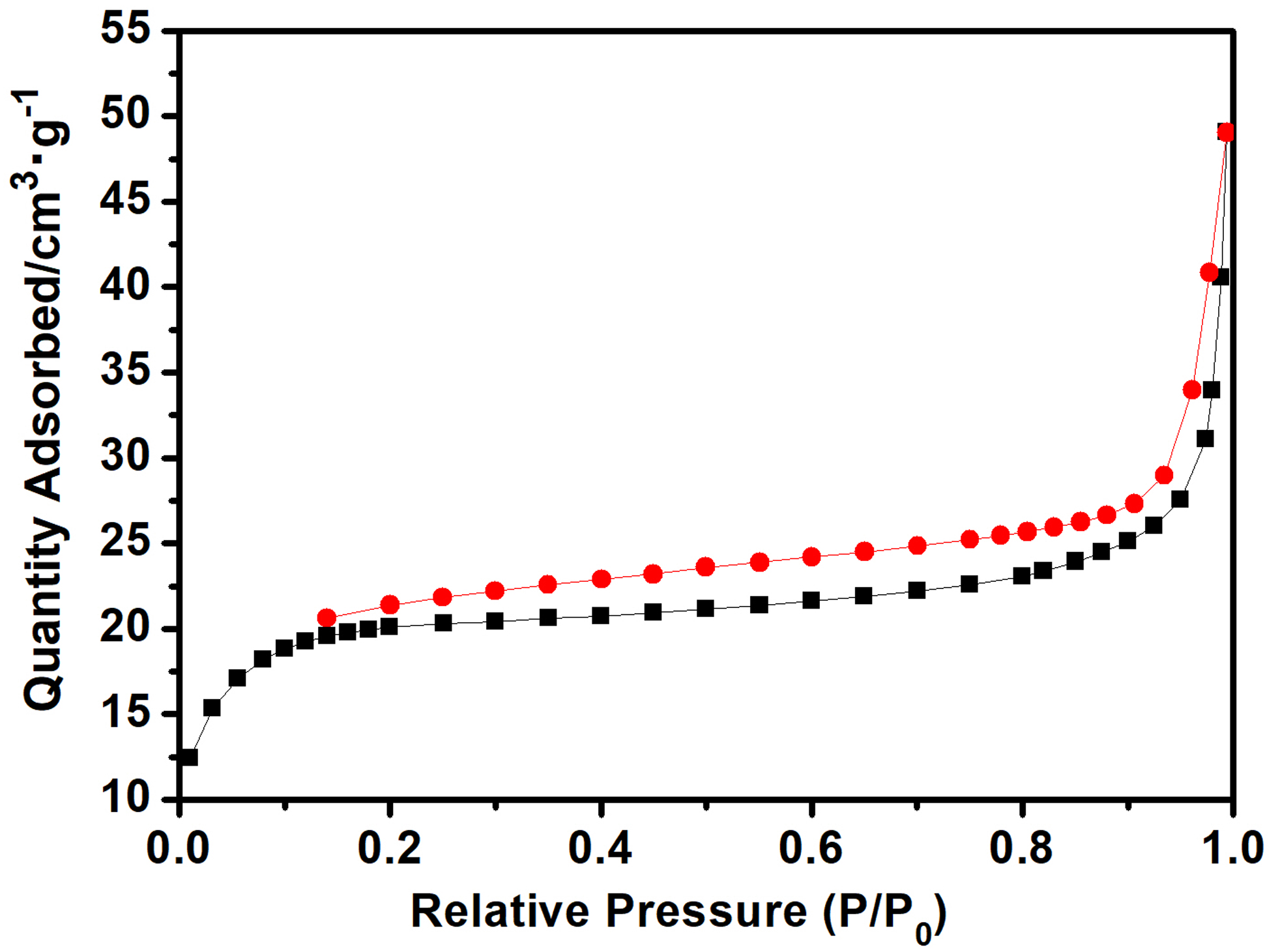
**

**Fig. S2** The typical N_2_ adsorption-desorption isotherm of SiO_2_@GdPO_4_:Tb@SiO_2_ nanoparticles.

**
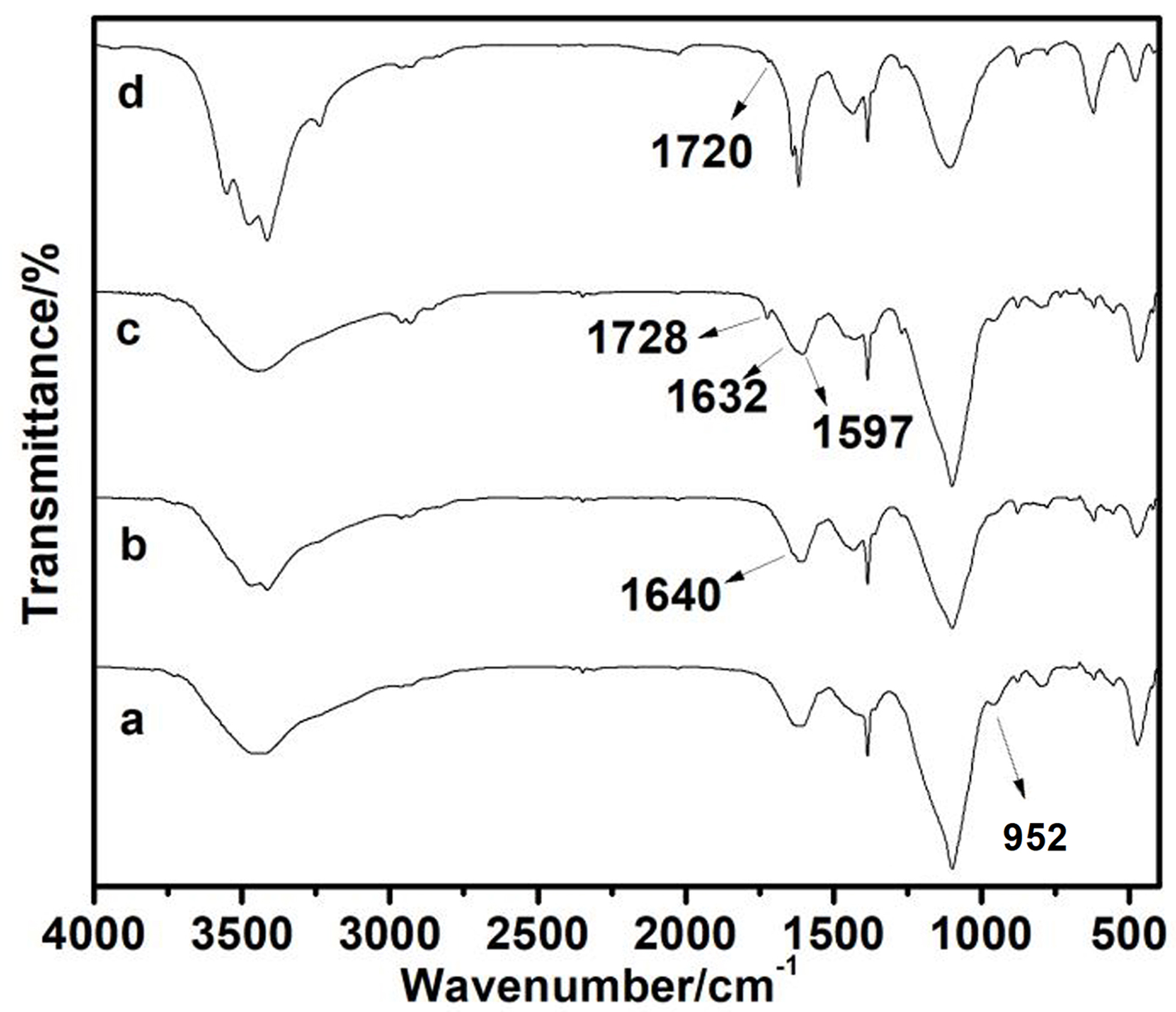
**

**Fig. S3** IR spectra of the products synthesized in different stages: (a) SiO_2_, (b) SiO_2_@NH_2_, (c) SiO_2_@MAH-Si, (d) SiO_2_@MAH-Si-Gd:Tb.


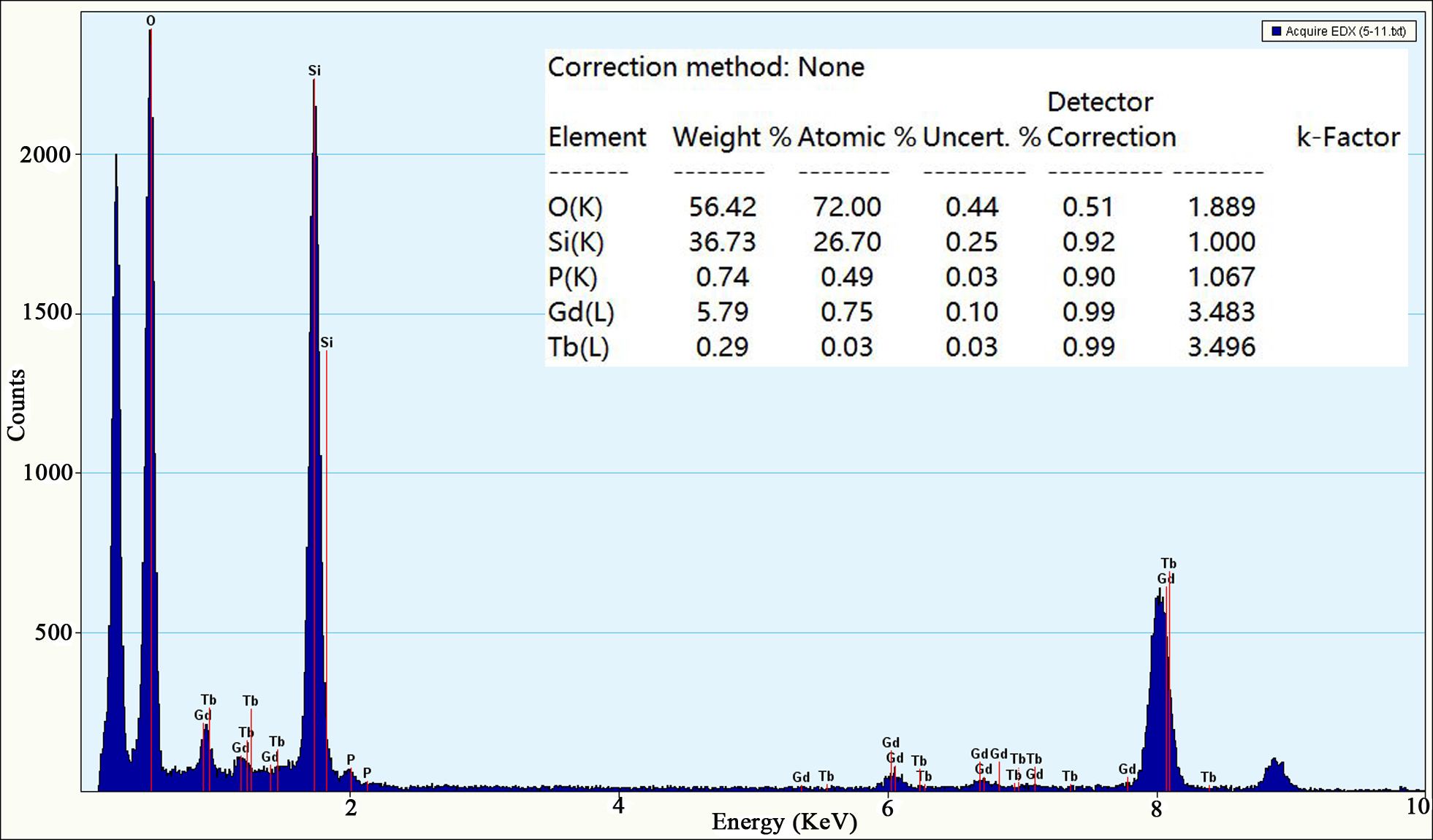


**Fig. S4.** Energy-dispersive X-ray spectroscopy of SiO_2_@GdPO_4_:Tb@SiO_2_ nanoparticles.


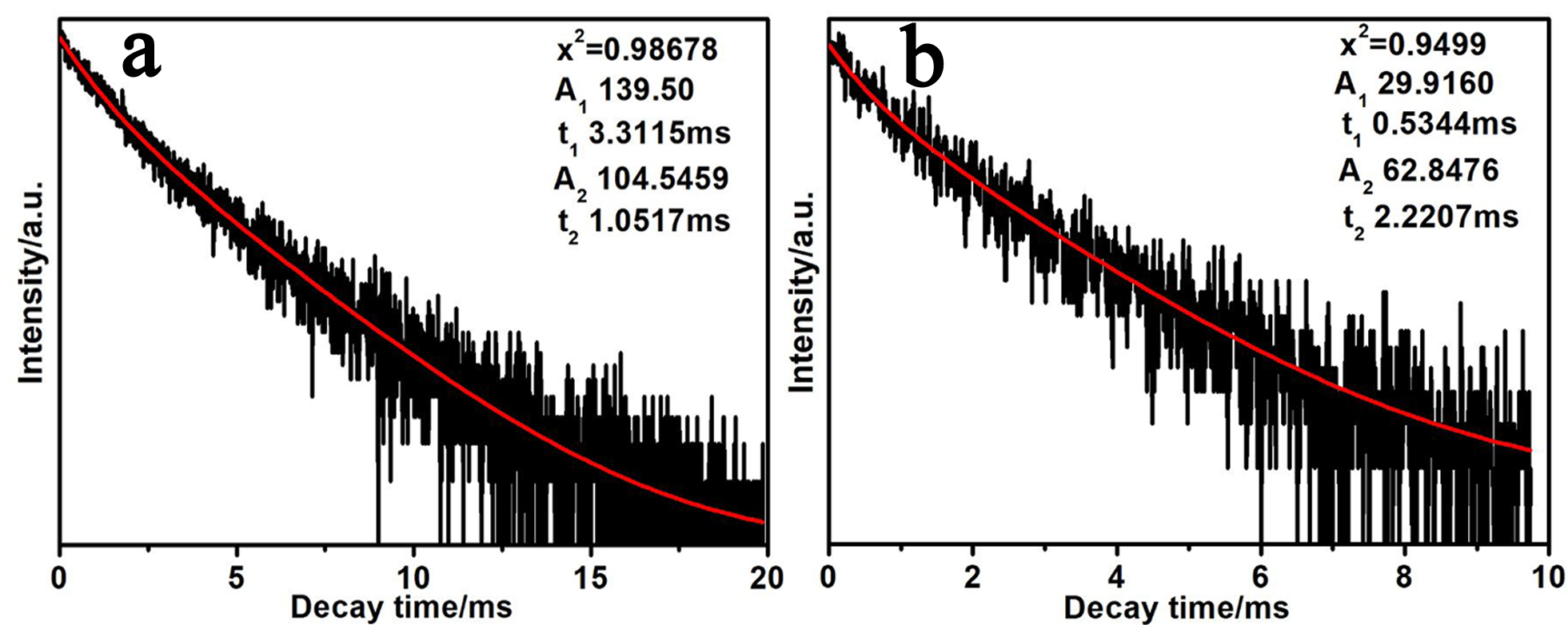


**Fig. S5** The fluorescence decay curve of (a) GdPO_4_:Tb nanoparticles (synthesized by hydrothermal method), (b) SiO_2_@GdPO_4_:Tb@SiO_2_ nanoparticles.
